# Genomic Analysis of Parent-of-Origin Allelic Expression in *Arabidopsis thaliana* Seeds

**DOI:** 10.1371/journal.pone.0023687

**Published:** 2011-08-17

**Authors:** Mary Gehring, Victor Missirian, Steven Henikoff

**Affiliations:** 1 Howard Hughes Medical Institute, Seattle, Washington, United States of America; 2 Fred Hutchinson Cancer Research Center, Seattle, Washington, United States of America; 3 Whitehead Institute for Biomedical Research, Cambridge, Massachusetts, United States of America; 4 Department of Biology, Massachusetts Institute of Technology, Cambridge, Massachusetts, United States of America; 5 Department of Computer Science, University of California Davis, Davis, California, United States of America; CNRS, France

## Abstract

Differential expression of maternally and paternally inherited alleles of a gene is referred to as gene imprinting, a form of epigenetic gene regulation common to flowering plants and mammals. In plants, imprinting primarily occurs in the endosperm, a seed tissue that supports the embryo during its growth and development. Previously, we demonstrated that widespread DNA demethylation at remnants of transposable elements accompanies endosperm development and that a subset of these methylation changes are associated with gene imprinting. Here we assay imprinted gene expression genome-wide by performing high-throughput sequencing of RNA derived from seeds of reciprocal intraspecific crosses. We identify more than 200 loci that exhibit parent-of-origin effects on gene expression in the endosperm, including a large number of transcription factors, hormone biosynthesis and response genes, and genes that encode regulators of epigenetic information, such as methylcytosine binding proteins, histone methyltransferases, and chromatin remodelers. The majority of these genes are partially, rather than completely, imprinted, suggesting that gene dosage regulation is an important aspect of imprinted gene expression.

## Introduction

The correct expression of imprinted genes, in which maternally and paternally inherited alleles are differentially expressed, is required for successful reproduction in both plants and animals [1]. Imprinted genes were initially identified in plants based on parent-of-origin effects on seed phenotypes [2] or through genetic screens aimed at identifying regulators of seed development [3], [4]. In plants imprinting occurs primarily in the endosperm, the seed tissue that nourishes the embryo. The embryo and endosperm are the twin products of double fertilization but differ in their ploidy; the embryo inherits one maternal and one paternal genome, whereas the endosperm inherits two maternal and one paternal genomes. Despite their genetic similarity and concurrent development, the embryo and endosperm are clearly epigenetically distinct [5]–[8].

Differential DNA methylation is an important aspect of the control of imprinted gene expression. For several imprinted genes the maternal allele is less methylated than the paternal allele in the endosperm [6], [9], [10]. Genome-wide DNA methylation mapping efforts further demonstrated that *Arabidopsis thaliana* endosperm is hypomethylated not just at imprinted genes but at thousands of sites throughout the genome when compared to the embryo and to vegetative tissues [6], [7]. Hypomethylation is primarily found at maternally-derived sequences. Similar results have been obtained for rice endosperm [11] and analysis of 5-methylcytosine content in maize indicates that endosperm is also hypomethylated in this species [12]. The difference in methylation between embryo and endosperm likely represents the outcome of multiple events, including active DNA demethylation in the female gamete that is the progenitor of the endosperm, decreased maintenance or *de novo* methylation during endosperm development, and/or increased methylation in the embryo [6], [7], [13]. Although methylation differences are found throughout the genome, only a subset of these likely impact gene expression.

Apart from the mechanistic basis of imprinted gene expression, parental conflict between maternally and paternally inherited genomes of offspring over maternal resource allocation is a popular explanation for why imprinted gene expression is evolutionarily advantageous (the parental conflict or kinship theory of imprinting) [14], [15]. Maternally expressed imprinted genes (MEGs) are expected to restrict offspring growth and paternally expressed imprinted genes (PEGs) are expected to promote growth. The theory fits well with the function of some of the known imprinted genes in plants; for example, *MEA* and *FIS2* are maternally expressed imprinted Polycomb group genes that restrict endosperm cell division. However, since the identity, functions, and expression patterns of many imprinted genes are likely still unknown it is presently unclear how many of the imprinted genes will reasonably fit under the umbrella of the kinship theory. Other theories suggests that in species where the mother provisions or cares for the offspring, expression of maternal alleles is favored due to an increase in the adaptive integration of maternal and offspring genomes (the maternal-offspring coadaptation theory of imprinting) [16]. More broadly, imprinted expression might be maintained at any locus that has dosage-dependent effects on seed viability [17].

We previously used knowledge of differences in methylation between *Arabidopsis thaliana* embryo and endosperm, as well as information on endosperm and developmental expression patterns [18], [19], to predict what genes were imprinted, five of which were validated by RT-PCR assays [6]. Our analysis of gene imprinting was restricted to those genes associated with methylation differences, but other epigenetic mechanisms, such as silencing mediated by Polycomb group (PcG) complexes, are also important for maintaining imprinted expression [9], [20], [21]. Relatively few large-scale unbiased screens of allelic expression patterns have been performed in plants. Allele-specific expression analysis in endosperm of reciprocal hybrids of maize indicates that most genes are expressed according to the contribution of the parental genomes, although a small proportion of the genes studied exhibited parent-of-origin specific expression patterns [22], [23]. The advent of high throughput RNA sequencing technologies makes it possible to more directly assess the relative quantities of steady-state transcripts derived from maternally or paternally-inherited alleles. Similar approaches have successfully identified genes imprinted during different stages of mouse development [24]–[26].

Here we assay imprinted gene expression by performing high throughput sequencing on poly-A selected RNA (RNA-seq) from embryo and endosperm derived from reciprocal crosses between two *Arabidopsis thaliana* accessions, L*er* and Col-0. This strategy allowed us to distinguish transcripts derived from the maternally inherited or paternally inherited allele for a portion of expressed genes with L*er*/Col-0 single nucleotide polymorphisms (SNPs). We identified >200 genes with parentally biased expression patterns. Our experimental strategy is particularly robust for identifying paternally expressed imprinted genes, as transcripts derived from the paternal genome must come from one of the fertilization products. Over 40 genes are predominantly paternally expressed, including a large number of transcription factors and chromatin related proteins. Most of the imprinted genes we identify exhibit parentally biased expression rather than complete monoallelic expression, suggesting that dosage regulation is an important factor in gene imprinting.

## Materials and Methods

### Plant Material and RNA Isolation

Wild type Col-0 and L*er* plants were grown in 16 hour days at 21° C. Stage 12c flowers [27] on four to five-week old plants were emasculated and pollinated two days later. Seeds were dissected into embryo, endosperm, and seed coat fractions as previously described at the torpedo stage of development [6], which under our growth conditions was at either 6 or 7 days after pollination (DAP) depending on the direction of the cross. Embryo and endosperm RNA was isolated using the RNAqueous Kit with Plant RNA Isolation Aid (Ambion), concentrated by precipitation with ammonium acetate, and treated with DNase I (Invitrogen). For L*er*×Col, RNA was pooled from dissected seeds from 21 siliques (∼800–1000 seeds). For Col×L*er*, RNA was pooled from seeds dissected from 14 siliques (∼560–700 seeds).

### High Throughput Sequencing

RNA-seq libraries were created by using the Illumina mRNA-seq kit and following the sample preparation guide protocol (Illumina). Briefly, 1 µg of total RNA was poly-A selected twice, fragmented, and converted into double-stranded cDNA. The cDNA was end repaired, adenylated, and adapters ligated. Adapter-ligated DNA approximately 200 bp in length was gel purified and amplified using Illumina PCR primers PE1.0 and PE2.0 with 15 amplification cycles. The library preparation method does not retain strand-specific information. Libraries were sequenced on two separate runs of Illumina GAII machines, including one lane each of Col×L*er* embryo and Col×L*er* endosperm and two lanes each of L*er*×Col embryo and L*er*×Col endosperm at 50 bp, and one additional lane of each library at 36 bp. Sequencing reads are deposited as fastq files in GEO record GSE30511 (http://www.ncbi.nlm.nih.gov/geo/query/acc.cgi?acc=GSE30511).

### Sequence Alignment

Sequencing reads were aligned to the TAIR 9.0 version of the Arabidopsis reference genome (Col-0) using TopHat [28]. Low quality reads and reads that mapped to more than one position in the genome with the same alignment score were discarded. Reads that mapped to more than one gene due to overlapping exons were not considered in further analyses. SAM files for each alignment are deposited in GSE30511.

### Assigning reads as Ler or Col

We used known SNPs from Perlegen and Ecker lab L*er* resequencing data downloaded from TAIR (ftp://ftp.arabidopsis.org/home/tair/Polymorphisms/; Table S1). Ecker SNPs where the L*er* consensus base was reported as occurring on fewer than 95% of base calls in L*er* were not used. For each read that overlapped a known Col/L*er* SNP we determined whether the read matched Col or L*er* at that position. If a read overlapped multiple SNPs it was only classified as Col or L*er* if all SNPs agreed.

### Detecting Imprinting

For each locus in the embryo or endosperm we determined a p-value for the null hypothesis of no imprinting and corrected for multiple testing using the Benjamini-Hochberg method. Specifically, we followed the method of Wang *et al.* (2008) [25] to test for a significant difference between *p1* and *p2* using the Storer-Kim method [29] as implemented in R (www.r-project.org) [30]. *p_1_* is defined as the Col portion for a given locus in Col×L*er* crosses, and *p_2_* is the portion of Col in L*er*×Col crosses. For the embryo we tested if *p_1_* = *p_2_* = 0.5 and for the endosperm if *p_1_* = 2*p_2_* = 0.67, because the ratio of maternal to paternal genomes in the endosperm is 2∶1. As read coverage increases smaller and smaller degrees of imprinting can be detected. We therefore computed an imprinting factor to determine the magnitude of imprinting. For each locus we determined the 95% confidence interval around the Col/L*er* read ratio from each sample. The imprinting factor is the low value of the high confidence interval divided by the high value of the low confidence interval for the reciprocal crosses. In essence, the imprinting factor is a variable where we have high confidence that the ratio of Col to L*er* reads is at least that many times greater in one of the reciprocal hybrids than in the other reciprocal hybrid.

### Detecting *cis* effects

In addition to imprinting effects, biased expression patterns can result from strain-specific effects on gene expression. For example, a Col allele might be more highly expressed than a L*er* allele, independent of the direction of the cross. We computed the p-value for the observed *cis-*effect given the null hypothesis of no Col vs. L*er* effect and corrected for multiple testing using the Benjamini-Hochberg method. The *cis* effect factor is computed in a similar manner to the imprinting factor.

### RPKM and Differential Expression

RPKM (reads per kb per million mapped reads) values were determined using the method of Mortazavi *et al* (2008) [31]. For transposable elements, only reads that mapped to transposable elements but did not overlap genic exons were considered. Differential expression between two experiments was determined by applying Fisher's exact test to the number of reads mapping uniquely to each locus, using the upper-quartile normalization method described in Bullard *et al* (2010) [32]. Specifically, the read count ratio for a given locus across two experiments was compared to the ratio of the 75^th^ percentile read count across the two experiments, where loci with zero read counts in both experiments were excluded when computing the 75^th^ percentile. This method has been shown to outperform total read count normalization [32]. The differential expression factor is determined in a similar manner to the imprinting and *cis* effect factors.

### Validation assays

Quantitative PCR (qPCR) was performed on the embryo and endosperm cDNA samples and the Illumina libraries to confirm enrichment for genes preferentially expressed in the embryo and endosperm and to determine if the sequencing libraries faithfully represented the original cDNA population. For qPCR, DNase I (Invitrogen) treated RNA from Col×L*er* embryo and endosperm samples was reverse transcribed with oligo dT using the Retroscript Kit (Ambion). qPCR was performed using a StepOne Plus qPCR machine and Fast SYBR Green reagent (Applied Biosystems). qPCR primers were designed using QuantPrime [33] and are listed in Table S12. Results were analyzed using the ΔΔ CT method [34]. Expression was normalized by actin 8 (AT1G49240). Three technical replicates were performed for each sample.

To assay AT3G03750/*SDG20* imprinting in embryos, oligo dT primed cDNA was created from the embryo RNA samples used to create the Illumina sequencing libraries and a set of independently isolated reciprocal F_1_ embryo RNA. *SDG20* was amplified with primers MG581 (5′-GCTGACCAGCTTATCAAGCAAGG-3′) and MG586 (5′-CCTTCTCCAAATCAGTAGAGCCGCTA-3′) for 40 PCR cycles. RT-PCR products were gel purified, cloned into the pCR 2.1 TOPO vector (Invitrogen), and individual clones subjected to dideoxy sequencing. Col/L*er* SNPs at TAIR9 chromosome 3 positions 941,455 (A to C) and 941,585 (G to C) indicated whether clones were derived from Col or L*er* alleles.

### Functional Analysis

Functional annotation of the 165 maternally expressed imprinted genes and 43 paternally expressed imprinted genes was performed separately to test for enrichments in GO_FAT terms, KEGG pathways, or INTERPRO domains using DAVID Bioinformatics Resources version 6.7 (http://david.abcc.ncifcrf.gov/) [35], [36]. The background set of genes for both analyses was the 10,316 endosperm-expressed genes with a Col/L*er* SNP and at least 15 informative reads. Categories with EASE scores (a modified Fisher's exact test used by DAVID) less than 0.01 are presented along with p-values obtained after correction for multiple testing by the Benjamini-Hochberg method.

## Results

### RNA-seq identifies new imprinted genes

**Figure 1:**
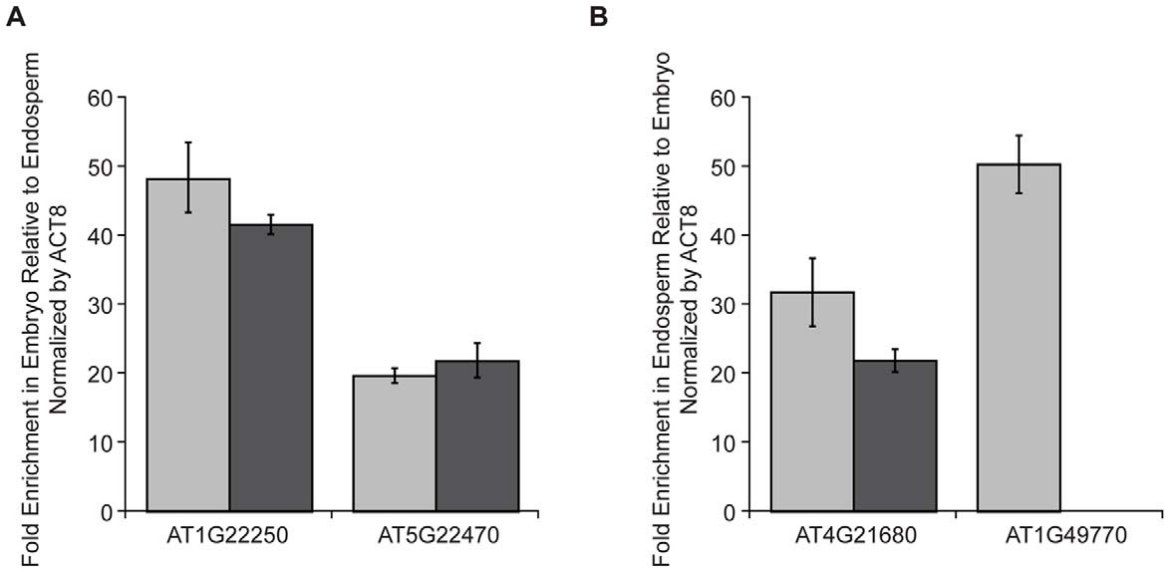
Embryo and endosperm specific genes are enriched in cDNA samples and resultant sequencing libraries. A) qPCR analysis of two genes known to be preferentially expressed in the embryo in Col×L*er* embryo cDNA (light gray bars) and sequencing libraries (dark gray bars). Values shown are fold enrichment in embryo relative to endosperm. B) qPCR analysis of two genes known to be preferentially expressed in the endosperm in Col×L*er* endosperm cDNA (light gray bars) and sequencing libraries (dark gray bars). Values shown are fold enrichment in endosperm relative to embryo. AT1G49770 could not be amplified from the Col×L*er* embryo sequencing library so enrichment could not be calculated. Data were normalized by ACT8 expression. Error bars represent standard deviation. AT1G22250 and AT4G21680 were identified as embryo or endosperm enriched genes in Col-0 microarray expression data (not shown). AT5G22470 has previously been shown by *in situ* analysis to be expressed in torpedo stage embryos [37] and AT1G49770 by *in situ* and reporter gene analysis to be expressed in the embryo-surrounding region of the endosperm [38], [39].

We performed a genomic analysis of imprinted gene expression in *Arabidopsis thaliana* embryo and endosperm to further understand the function, mechanisms, and evolution of gene imprinting. Wild type Col-0 females were crossed to L*er* males and L*er* females to Col-0 males. Seeds were dissected into their component parts at the torpedo stage of seed development. Four high throughput mRNA sequencing libraries were generated from reciprocal F_1_ hybrid Col/L*er* embryo and endosperm. qPCR on four genes known to be either preferentially expressed in the embryo (AT1G22250 and AT5G22470) or endosperm (AT4G21680 and AT1G49770/*RGE1*/*ZOU*) [37]–[39] indicated consistent enrichment between cDNA and sequencing library samples (Figure 1). We obtained between 34 and 41 million reads from each of the four libraries, 79% of which mapped uniquely to the genome using the TopHat read aligner [28]. 94% of those reads mapped to known genes. The direction of the cross (Col×L*er* vs. L*er*×Col) had only minor effects on gene expression, with high correlations between embryo samples and between endosperm samples (Pearson's *r* for RPKM values = 0.975 and 0.998, respectively). Embryo and endosperm expression profiles were clearly distinct (Pearson's *r* for Col×L*er* RPKM values = 0.144).

**Figure 2:**
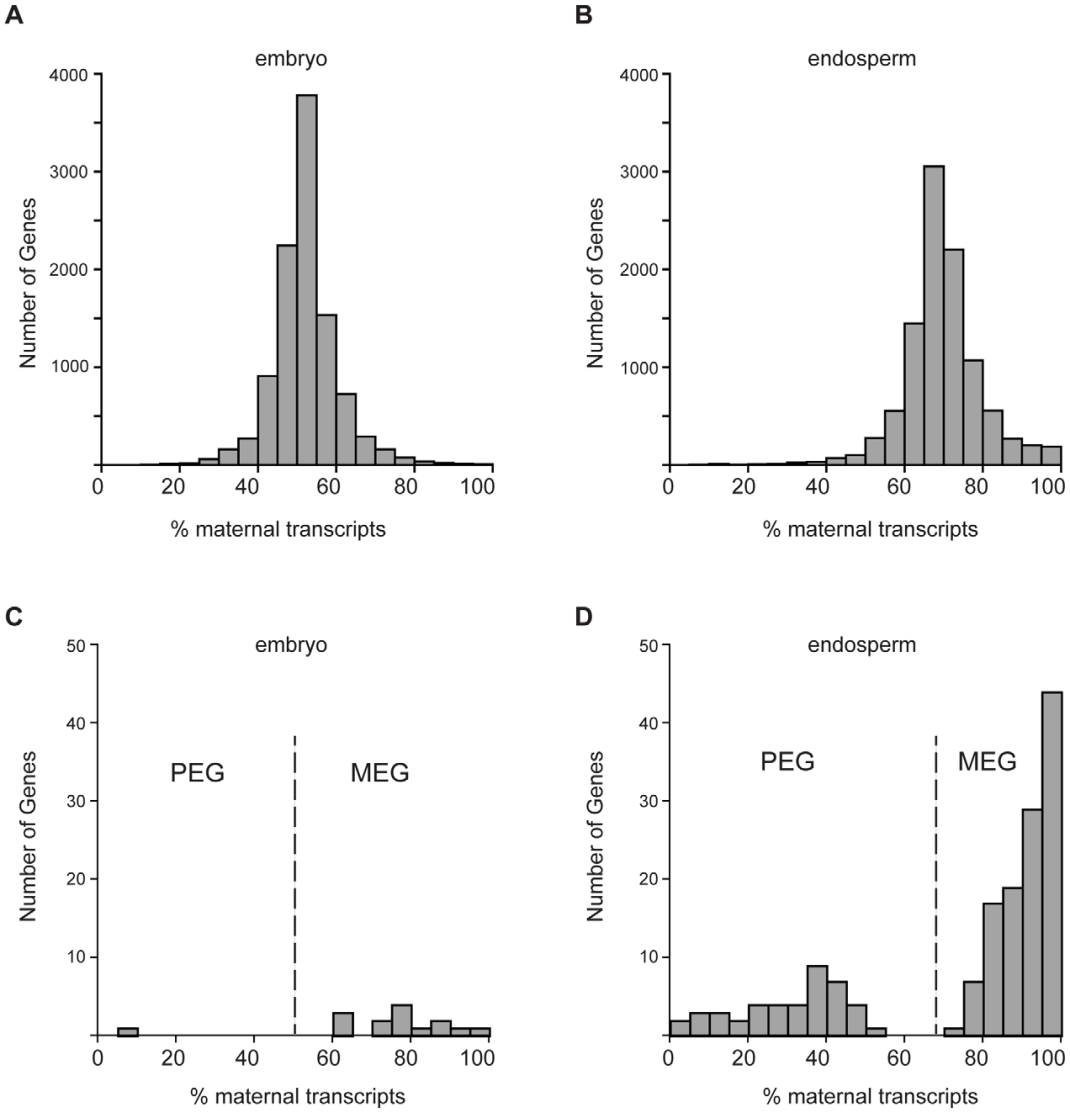
Hundreds of Arabidopsis genes exhibit parentally biased expression. A) Percent maternal transcripts from 10,340 genes expressed in the embryo with at least 15 informative reads. B) Percent maternal transcripts from 10,316 genes expressed in the endosperm with at least 15 informative reads. C) Percent maternal transcripts of 18 genes in the embryo that met imprinting criteria. D) Percent maternal transcripts of 208 genes in the endosperm that met imprinting criteria. MEG, maternally expressed imprinted gene; PEG, paternally expressed imprinted gene.

**Figure 3:**
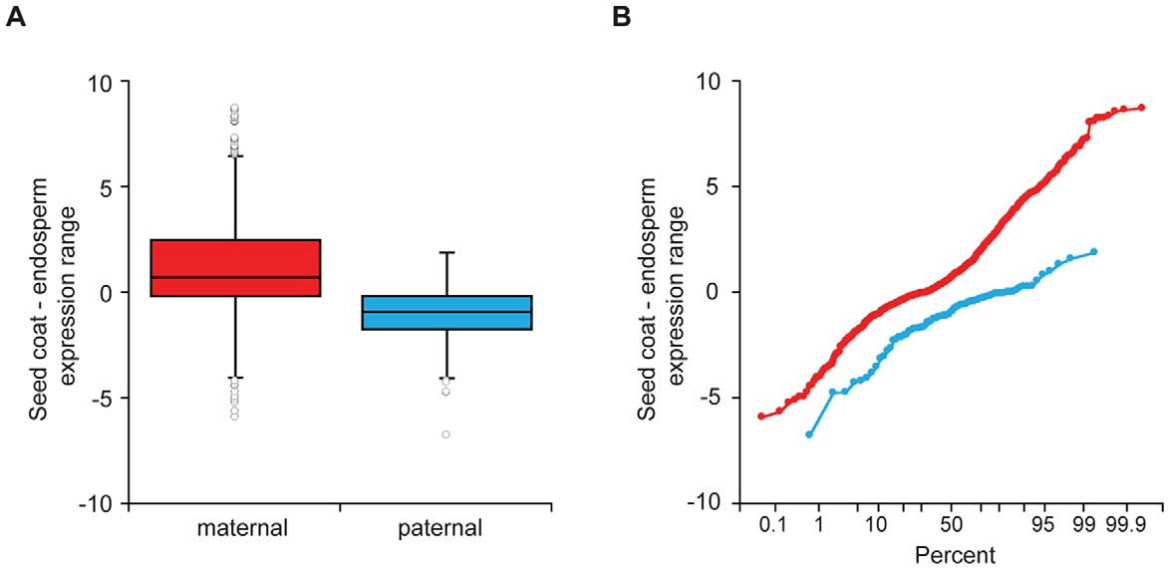
Designation of maternally expressed imprinted genes could be affected by maternal seed coat contamination. A) Box plots of seed coat – endosperm expression differences (log_2_) calculated using data from Le *et al.* [40] for genes exhibiting parentally biased expression in the endosperm at a p-value<0.01. B) Probability plots of the same data. Red, potential MEGs; blue, potential PEGs.

Although all mapped reads can be used to determine overall gene expression levels, only a fraction of the reads are informative for determining maternal or paternal allele expression – those reads that overlap a Col/L*er* SNP. Data from reciprocal crosses also allows parent-of-origin effects to be distinguished from strain specific biases in gene expression. We used information on previously identified SNPs (Table S1) to identify reads as Col or L*er*. We obtained between 1.56 and 1.96 million informative reads for each library and calculated the number of Col or L*er* reads for each gene. Approximately 10,300 genes from each tissue had at least 15 informative reads when data from reciprocal crosses were combined. These genes exhibited a range of maternal to paternal expression ratios, but the average percent maternal transcripts for each gene in the embryo and endosperm was near the expectation of 50% and 67%, respectively, based on the genomic DNA content of each tissue (Figure 2). This is consistent with studies of maize endosperm, which show that expression is proportional to the genomic contribution of the parents for most genes [22], [23]. To identify imprinted genes we used the Storer-Kim method [29] to test whether the proportion of maternal and paternal reads for each gene was significantly different from expectations [25], taking into account the allele-specific read counts from both reciprocal crosses in order to distinguish parent-of-origin effects from strain-specific effects. We initially considered genes with a p-value less than 0.01, identifying 148 potential imprinted genes in the embryo (142 maternally expressed imprinted genes or MEGs, 6 paternally expressed imprinted genes or PEGs) and 1437 in the endosperm (1334 MEGs, 103 PEGs). Five of the 11 previously identified imprinted genes passed this initial p-value cutoff, while the remaining known imprinted genes either lacked L*er*/Col SNPs, had very few informative reads, or, in one case, fell just below the cutoff (Table 1). Allele-specific expression data, RPKM values, and embryo-endosperm differential expression analysis for all genes is presented in Tables S2 and S3. There was very little evidence for expression or imprinting of transposable elements in either tissue (Tables S4 and S5). Upon closer examination of some of the most highly parentally biased genes, it was clear that transcripts from genes highly expressed in the seed coat (which is diploid maternal tissue) were contaminating both the embryo and endosperm fractions. Some contamination from abundant seed coat transcripts is likely unavoidable given our method of seed dissection, which is performed in an aqueous solution on a glass slide. We used expression information from another seed gene expression set to further filter our data. Le *et al.* (2010) [40] used laser capture microdissection (LCMD) to isolate tissue from embryo, endosperm, and seed coat in the Ws background and determined gene expression values using Affymetrix microarrays. The rank order correlation coefficient between our embryo or endosperm expression data and the LCMD gene expression data of tissues at the same stage of development was good (*r* = 0.81 for embryo and *r* = 0.77 for endosperm). To determine a reasonable cutoff for removing genes likely to be affected by maternal seed coat contamination, we examined the difference between LCMD seed coat and endosperm expression for the 82 potential endosperm PEGs with a p-value less than 0.01 and Affmetryix expression data. Maternal seed coat contamination cannot result in false positive PEGs, only false negatives. The average difference in seed coat and endosperm expression was −1.1 for potential endosperm PEGs and 1.2 for potential MEGs (Figure 3). The maximum PEG difference was 1.93, but 95% of the genes had a seed coat-endosperm value less than 1.04 (Figure 3). We thus removed genes from the pool of potential MEGs that had approximately two fold higher expression in the seed coat than endosperm (a difference of 1.04 between GCRMA normalized expression values), although we retained genes that lacked Affymetrix data. The same filtering was performed on the embryo dataset. This reduced the number of potential imprinted genes to 905 in the endosperm (802 MEGs, 103 PEGs) and 87 in the embryo (81 MEGs, 6 PEGs).

**Table 1:** RNA-seq results for previously identified imprinted genes.

		Col×L*er* endosperm				L*er*×Col endosperm					
Gene ID	Name	Reads	RPKM	Col	Ler	Reads	RPKM	Col	Ler	p-value	Bias
AT1G02580	*MEA*	14	0.2	0	0	21	0.3	0	0	-	No SNP
AT1G65330	*PHE1*	21	0.9	0	0	17	0.7	0	0	-	No SNP
**AT2G32370**	***HDG3***	**3735**	**50.5**	**79**	**190**	**4857**	**56.2**	**303**	**96**	**0**	**Paternal**
AT2G35670	*FIS2*	212	3.2	13	0	355	4.6	6	16	3.59e-03	Maternal
AT3G03260	*HDG8*	53	0.9	2	6	69	0.9	12	8	1.68e-02	Few reads
AT3G19350	*MPC*	6	0.4	0	0	30	1.8	0	0	-	No SNP
**AT4G00540**	***MYB3R2***	**377**	**9.5**	**129**	**0**	**264**	**5.7**	**9**	**77**	**0**	**Maternal**
AT4G25530	*FWA*	113	1.8	5	0	54	0.7	0	0	1	Few reads
**AT5G17320**	***HDG9***	**869**	**15.6**	**29**	**1**	**1775**	**27.3**	**3**	**117**	**1.44e-20**	**Maternal**
AT5G54650	*FH5*	538	6.1	54	12	495	4.8	14	43	5.08e-03	Maternal
AT5G62110		42	0.7	1	3	31	0.5	2	0	3.76e-01	Few reads

Genes in bold pass all of the imprinting criteria used in this study.

As read coverage increases it is possible to detect smaller and smaller degrees of imprinting with statistical significance. Genes with a large number of informative reads can have very low p-values, even if the parental bias is intuitively not very strong. For example, locus AT2G05990 has 15,636 informative reads, 37% of which are paternally derived (the expectation for paternal reads is 33.3%). This gene is identified as paternally biased with a p-value of 3.65×10^−21^. Thus, by p-value considerations alone, AT2G05990 would be considered imprinted. Therefore, to describe the strength of imprinting in a manner less dependent on read depth, we also calculated an imprinting factor for each locus (see Methods) and further restricted our analysis to genes with an imprinting factor of at least 2, meaning that the ratio of Col to L*er* reads in one cross was at least 2 fold different from the Col/L*er* ratio in the reciprocal cross. By these criteria, AT2G05990 is discarded because the imprinting factor is 1.25 (Table S2). We also removed a few genes with strong *cis* effects on expression (an allele from one strain was dominant, independent of the parent-of-origin). After these final filtering steps we identified 18 genes with biased expression in the embryo (17 MEGs, 1 PEG) and 208 genes in the endosperm (165 MEGs, 43 PEGs) (Figure 2; Tables S6 and S7). The endosperm list includes three previously identified imprinted genes: *HDG3*, *HDG9*, and *MYB3R2* (Table 1) [6]. The list does not include the known imprinted gene *FIS2*, which passed our p-value threshold but has a low imprinting factor due to low read counts. We are likely missing other valid imprinted genes on our list (false negatives). Increasing sequencing depth could reduce false negatives.

Although all genes in our list pass the same statistical criteria, we consider the identification of PEGs more robust than the identification of MEGs. The MEG list is more likely to contain false positives than the PEG list because any contamination from maternally derived tissues will make genes appear more maternally expressed than expected. The only source of the paternal genome is from the products of fertilization, embryo and endosperm, and thus contaminating RNA from maternally derived tissues cannot create false positives, only false negatives.

The data indicate that imprinting is primarily endosperm-specific at this stage of seed development. One embryo-imprinted locus has been identified in maize [41] but whether this represents a more widespread phenomenon is unknown. The 18 genes we identify as imprinted in the Arabidopsis embryo (Table S7) may represent the rate of false positives in our experiments. Fourteen of the 17 MEGs are more highly expressed in endosperm than in embryo and 7 are also identified as MEGs in the endosperm. This could indicate cross contamination of embryo with endosperm RNA. Further experimentation and validation using reporter genes or *in situ* analysis will be required to conclusively determine if these genes truly exhibit parent-of-origin specific expression in the embryo. One embryo gene, *SDG20*, was identified as a PEG, which could not be due to maternal contamination. We attempted to independently validate paternally biased expression of this gene by performing RT-PCR on the original RNA samples used to create the embryo libraries and on a set of independently isolated F_1_ RNA. After sequencing cloned RT-PCR products, we identified 15 Col clones and 11 L*er* clones from the original Col×L*er* F_1_ embryo sample and 17 Col clones and 7 L*er* clones from the original L*er*×Col F_1_ embryo sample. Independently isolated samples had 18 Col clones and 11 L*er* clones in a Col×L*er* cross, and the reciprocal indicated 14 Col clones and 14 L*er* clones. Combining the data, in each F_1_ genotype 60% of the reads are derived from the Col allele, indicating a bias towards the Col allele independent of the parent of origin. The bias was also observed in dCAPS analysis of RT-PCR products (data not shown). Therefore, *SDG20* is not imprinted in the embryo. We consider it likely that all of the 18 embryo genes are false positives.

### Partial imprinting is more common than complete imprinting

Most endosperm-imprinted genes (140 of 208) are more highly expressed in the endosperm than in the embryo (Table S6), which is consistent with the idea that imprinted genes are involved in endosperm specific functions. However, it is important to note that most of the genes identified as imprinted in our quantitative assay exhibit partial rather than complete imprinting (also referred to as differential and binary imprinting [17]). Genes that exhibit partial imprinting are differentially expressed in a parent-of-origin specific manner, but do have transcripts derived from both alleles. Similar results have recently been obtained from a study of imprinting in mouse brains, where most imprinted genes do not exhibit strict monoallelic expression [26]. Partial imprinting is typical for PEGs but relatively rare for MEGs; only six of the PEGs exhibit >90% paternal transcripts (Figure 2, Table S6). In contrast, 121 MEGs have greater than >90% maternal transcripts (Figure 2, Table S6), with the caveat that any contamination from maternal tissue will tend to make PEGs look partial and MEGs complete. Whether or not the mechanisms of imprinting and selection pressures acting at partially and completely imprinted genes are the same is unknown.

### Paternally expressed imprinted genes encode potential regulators of the epigenome

We previously suggested that imprinted genes were enriched for transcription factors and chromatin related proteins [6]. Gene ontology analysis indicates that these genes are prominent, although not the most highly enriched, among MEGs and PEGs. Maternally expressed imprinted genes included transcription factors such as *HDG9*, *MYB115*, *MYB77*, *MYB3R2*, *NF-YC12*, and several zinc finger and leucine zipper genes. Additionally, MEGs were strongly enriched for genes involved in cell wall modification, particularly pectinesterases (Table 2). Paternally expressed imprinted genes also included putative transcription factors and DNA binding proteins, such as *HDG3*, another homeodomain like gene, and genes containing ARID/BRIGHT DNA binding domains (Table S6). In addition, we identified 8 PEGs with potential roles in epigenetic regulation, including 3 members of a 5-methylcytosine binding gene family, a putative SNF2-related chromatin remodeler, a histone deacetylase interacting protein, two SRA domain-containing histone lysine methyltransferases, and the PolIVa RNA polymerase (Table 3). The most strongly imprinted paternally expressed gene is *VIM5* (Table 3, Table S6), which is a member of a 6-gene family of SRA-domain containing 5-methylcytosine binding proteins. The founding member of the *VIM* family, *VIM1*, was identified as a loss of function mutant that caused centromere hypomethylation [42]. *VIM1*, *VIM2*, and *VIM3* act redundantly to maintain CG methylation at genic and heterochromatic sequences [43], [44]. Efforts to find transcripts associated with *VIM5* were previously unsuccessful, and it was hypothesized that *VIM5* might be a pseudogene [43], [44]. However, our data show that *VIM5* is expressed specifically in the endosperm almost entirely from the paternal allele. *VIM1*, which is adjacent to *VIM5* on chromosome 1, and *VIM6/ORTHL1*, are also PEGs, although imprinting is only partial, and both genes are more highly expressed in the embryo than endosperm (Table 3, Table S6). The function of any of the potential epigenetic regulators during seed development is presently unknown.

**Table 2:** Functional enrichments of imprinted genes.

MEGs				
Type	Term	Fold Enrichment	EASE p-value	Benjamini p-value
Interpro domain	pectin lyase fold	10.2	2.7e-4	6.1e-2
Interpro domain	pectinesterase inhibitor	9.4	1.8e-3	0.19
Biological Process	response to wounding	7.1	1.4e-3	0.46
Biological Process	external encapsulating structure	5.4	4.4e-3	0.48
Molecular Function	pectinesterase activity	9.4	3.8e-4	6.7e-2
Molecular Function	transcription factor activity	2.3	2.6e-3	0.11
Molecular Function	carboxylesterase activity	4.3	5.2e-3	0.17
Cellular Component	endomembrane system	1.9	6.4e-4	4.2e-2
Cellular Component	plant cell wall	4.5	1.8e-3	6.0e-2
Cellular Component	anchored to membrane	3.9	8.2e-3	0.17

PEGs				
Type	Term	Fold Enrichment	EASE p-value	Benjamini p-value
Interpro domain	SRA-YDG	106.7	8.0e-8	6.3e-6
Biological Process	cotyledon development	65.4	8.1e-4	0.14
Biological Process	chromatin modification	13.3	2.6e-3	0.22
Biological Process	regulation of transcription	3.5	3.4e-3	0.19

MEGs and PEGs associated with DMRs				
Type	Term	Fold Enrichment	EASE p-value	Benjamini p-value
Biological Process	regulation of transcription	3.9	1.0e-5	1.0e-3
Biological Process	cotyledon development	41.4	2.1e-3	0.15
Biological Process	chromatin organization	7.4	3.7e-3	0.20
Molecular Function	DNA binding	2.4	9.5e-4	5.1e-2
Molecular Function	transcription factor activity	3.1	3.2e-3	0.11

**Table 3:** Paternally expressed imprinted genes that encode potential regulators of the epigenome.

Locus ID	Gene name	Endosp RPKM	Maternal Reads	Paternal Reads	% paternal	Imprinting p value	Imprinting Factor	Gene Description
AT1G57800	*VIM5*	58.2	142	1898	93.0	0	478.81	putative 5-methylcytosine binding protein
AT1G57820	*VIM1*	17.6	181	302	62.5	2.57e-33	7.31	5-methylcytosine binding protein; ubiquitin E3 ligase
AT4G08590	*VIM6*	15.4	99	147	59.8	2.15e-12	3.89	putative 5-methylcytosine binding protein; ubiquitin E3 ligase
AT1G17770	*SUVH7*	0.95	10	32	76.2	2.29e-05	5.08	histone lysine methyltransferase
AT4G13460	*SUVH9*	12	86	130	60.2	6.41e-10	3.54	histone lysine methyltransferase
AT2G21450	*CHR34*	7.3	13	43	76.8	1.41e-06	7.56	SNF2 family chromatin remodeling helicase
AT1G63020	*PolIVa*	10.2	168	207	55.2	2.50e-15	3.45	Plant specific RNA polymerase involved in RNA-directed DNA methylation
AT1G59890	*SNL5*	3.1	14	28	66.7	2.23e-03	2.77	SIN3-like5; transcriptional regulator associated with histone deacetylases

Genes involved in hormone biosynthesis or perception have not previously been implicated as imprinted genes but were featured in both our list of MEGs and PEGs. We identified as MEGs *OPR3*, a gene involved in jasmonate (JA) biosynthesis, a zinc finger transcriptional repressor, *STZ/ZAT10*, that regulates JA biosynthesis genes, *BR6OX*, whose product generates the active form of brassinosteriods, and two genes involved in ethylene biosynthesis and response, *EIN2* and *MKK9*. In addition, two auxin biosynthesis genes, *YUC10* and *TAA1*, are paternally expressed imprinted genes (Table S6) [45]–[47]. Another MEG, *JLO*, has been shown to promote expression of auxin-efflux carriers [48], [49]. Both *JLO* and *YUC10* have important roles in embryo patterning during seed development [47]–[49]. Although many of these genes have been extensively studied in other contexts, our results suggest that multiple hormone pathways may have as yet unappreciated roles in endosperm development and function.

### Imprinted genes that encode regulatory proteins are associated with differential DNA methylation

Our comprehensive survey of gene imprinting allowed us to assess the congruence of gene imprinting with other features of the genome. We previously analyzed methylation differences between embryo and endosperm and were able to identify new imprinted genes by identifying genes associated with lower methylation in the endosperm than embryo, preferential expression in the endosperm, and low expression in other tissues [6]. Loss of methylation primarily occurred on repetitive sequences derived from transposable elements (TEs). We designated ∼50 genes as likely imprinted genes based on these characteristics [6]. Our RNA-seq data indicates that several of these genes are indeed imprinted. Twenty of the candidate genes have sufficient read coverage and SNPs to assay imprinting, 11 of which pass our initial p-value threshold for imprinting. Four genes pass all of our criteria for imprinting (Table S8).

We examined the overlap between the 208 imprinted genes identified by RNA-seq and the top positive embryo-endosperm differentially methylated regions (DMRs) (previously identified regions of endosperm hypomethylation in the top 0.5% of differences) [6]. 63 of the endosperm imprinted genes harbor a top Col-*gl* and/or L*er* DMR within the gene or 2 kilobases 5′ or 3′ (Table S6). This is almost 3-fold higher than the association between the same number of randomly selected informative genes and DMRs (n = 24) and represents a significant enrichment (Fisher's exact test p<0.0001). Many of these genes are also more highly methylated in demethylase deficient endosperm than in wild type endosperm (Table S6). The association between DMRs and gene imprinting is particularly strong for the PEGs, where half of the genes are associated with DMRs (22/43). All of the PEG potential epigenetic regulators (Table 3; Table S6) are associated with DMRs. Many of the MEGs associated with DMRs encode transcription factors, as well as some of the genes involved in ethylene, jasmonate, and brassinosteriod biosynthesis and/or perception (Table S6). Overall, the imprinted genes associated with DMRs are enriched for the GO term “regulation of transcription” (Table 2).

In addition to DNA methylation, chromatin based silencing mechanisms mediated by Polycomb group complexes (PcG) are important for maintaining imprinted gene expression. These two mechanisms can act independently or in concert at a locus. The PcG group complex consisting of FIE/FIS2/MEA is required to maintain imprinted gene expression at several loci, including *PHE1* and *MEA* [9], [20], [21], which are also associated with DMRs. The Polycomb group complex methylates lysine 27 on histone H3, a chromatin modification associated with stable states of gene repression. We compared our set of imprinted genes to genes that contain H3K27me3 during early endosperm development (1–4 DAP) as described by Weinhofer *et al.* [50]. Twenty-one imprinted genes are associated with H3K27me3 in the endosperm. Among these are five genes also associated with DMRs, including *HDG3*, *HDG9*, and *SUVH7* (Table S6). We also examined the congruence between our dataset and gene expression in whole *fis2* seeds at 3 and 6 DAP [50] and siliques from *mea/fis1* mutant females crossed to wild type males [51]. Imprinted genes where one allele is repressed by the PcG complex might be overexpressed in PcG mutants. Two genes were upregulated in both *fis2* and *mea/fis1* (Table S6), the PEGs *SUVH7* and *FXG1*, an alpha-fucosidase involved in cell wall metabolism [52]. *SUVH7* is the only imprinted gene identified as both associated with H3K27me3 and upregulated in the PcG mutants – we thus consider *SUH7*, which itself encodes a histone methyltransferase, an excellent candidate for an imprinted locus directly regulated by the PcG complex.

### Imprinted genes are not extensively clustered

**Figure 4:**
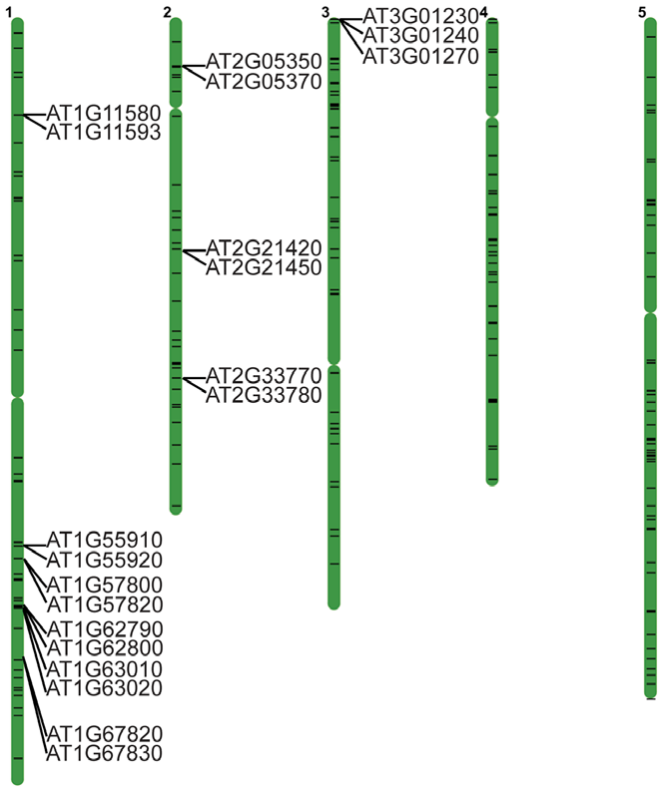
Some imprinted genes are found in mini-clusters. Position of endosperm imprinted genes (horizontal black lines) on the five Arabidopsis chromosomes. Imprinted genes within 10 kb of one another are listed. Positions were mapped using the chromosome map tool on TAIR (www.arabidopsis.org).

In mammals imprinted genes often lie in clusters and expression is controlled by an imprinting control region (ICR) [53]. We compared the distance between imprinted genes, with the caveat that imprinting can only be assessed for the subset of genes with a L*er*/Col SNP and sufficient read coverage. The average distance between imprinted genes was not significantly different from the average distance between randomly selected informative genes. However, we identified 10 regions in the genome where two or three imprinted genes were within 10 kb of one another, including 6 instances in which adjacent genes were imprinted (Figure 4). These mini-clusters might represent genes controlled by common *cis* epigenetic regulatory elements.

### Comparison to other genome-wide imprinting data

**Figure 5:**
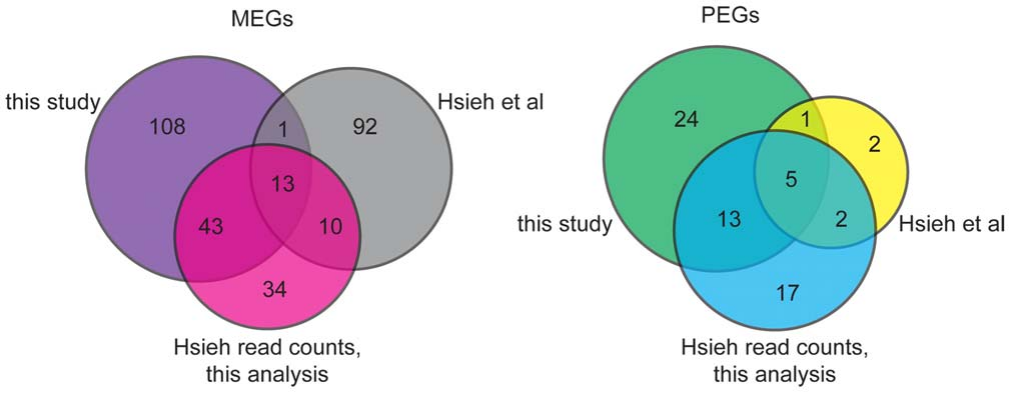
Overlap among Arabidopsis imprinted genes identified in different studies increases when the same analysis methods are applied. Venn diagrams compare overlap among maternally and paternally expressed endosperm imprinted genes identified in this study (Table S6), in Hsieh *et al.* [54], and when Hsieh *et al.* read counts are analyzed using the analysis developed in this study.

Hsieh *et al.* [54] recently published results from a similar experiment, combining data from manually dissected endosperm and LCMD endosperm, to analyze imprinted gene expression in Col/L*er* reciprocal crosses. Using different statistical analyses and criteria, they identified 116 MEGs and 10 PEGs in the endosperm. Fourteen of the MEGs are in common with the 165 MEGs we identified, as are 6 PEGs (Figure 5 and Table S9). While the overlap between datasets is low, much of this is due to statistical cutoffs and read coverage. In our dataset, 42 of their 116 MEGs passed our initial p-value cutoff of less than 0.01, but many genes were then discarded because of failure to meet our other two criteria. Additionally, 27 of their MEGs had too few reads in our dataset to assess imprinting. All four of the Hsieh *et al.* PEGs that were not found in our list have very low read numbers in our dataset (less than 10 total reads), although almost all of these reads are paternal. We also analyzed their Col/L*er* read counts for each gene using our statistical pipeline. The highest overlap between our set of imprinted genes was with their LCMD endosperm dataset. Applying our pipeline to their read counts, we find 137 imprinted genes in their dataset (100 MEGs, 37 PEGs), more than half of which (n = 74) are in common with our set of 208 imprinted genes (56 MEGs, 18 PEGs) (Figure 5, Tables S10 and S11). A consideration of both datasets, along with additional experimental validation, will likely be most robust for identifying valid imprinted genes.

## Discussion

We have used high-throughput mRNA sequencing to identify genes imprinted in *Arabidopsis thaliana* endosperm. Our analysis identified dozens of new imprinted genes involved in transcriptional regulation, epigenetic processes, hormone biosynthesis and reception, and cell wall function. The function of most of these genes during seed development is unknown and the data represent a rich source for further understanding endosperm development, the mechanisms of gene imprinting, and the selection pressures driving its evolution.

While we have performed a genomic analysis of gene imprinting, it is important to emphasize that our list of imprinted genes is not comprehensive. Some known imprinted genes, like *MEA*, *FWA*, and *PHERES1*, did not arise in our analysis because of lack of SNPs or low expression. Furthermore, our list is specific to a particular stage of seed development, and we expect that different sets of imprinted genes are active at earlier stages. Moreover, it should be noted that our assay necessarily only reports on steady-state transcript levels, which could be impacted by a number of processes in addition to transcription itself, including maternal deposition of RNAs (although our profiling takes place several days after fertilization), transport of RNAs from other tissues, and transcript degradation. The most stringent test of imprinting is to show that transcription itself is differential between alleles [55], which our method cannot address.

Our initial analysis of gene imprinting based on DNA methylation profiling discovered a subset of imprinted genes associated with TE-derived differentially methylated regions [6]. Our present survey shows that about a third of imprinted genes, often encoding regulatory proteins, are associated with differential DNA methylation within 2 kb of the coding sequence. However, a number of MEGs, many of which encode enzymes like pectinmethylesterases, pectinesterase inhibitors and glycosyl hydrolases, all involved in cell wall modification, are not associated with DMRs. The mechanism of parentally biased expression at these loci likely does not directly involve DNA methylation.

Striking features of our data are that most genes exhibit partial imprinting rather than strict monoallelic expression and that MEGs are more numerous than PEGs. The kinship theory of imprinting predicts that monoallelic expression (one allele expressed, one allele completely silent) is the evolutionary stable strategy for genes in which the maternally and paternally derived alleles favor different optimal levels of expression [15]. If not due to conflict, why do so many *Arabidopsis thaliana* genes exhibit parent-of-origin specific biased expression patterns? One possibility is that partial imprinting is an evolutionary echo of complete imprinting that existed at these genes when *A. thaliana* possessed a different mating system. *A. thaliana* is primarily self-fertilizing, with low but variable rates of outcrossing observed in the wild [56], [57]. Because maternal and paternal genomes are usually identical, conflict is expected to be very low in *A. thaliana* seeds (although in the crosses used in this experiment the maternal and paternal genomes are genetically distinct). However, *A. thaliana* is estimated to have been self-fertilizing for a short amount of evolutionary time – perhaps only 400,000 years [58], [59]. Furthermore, despite the loss of genetic conflict, as a mating system shifts from outcrossing to selfing, loss of imprinting is not predicted to be rapid [15]. Genes that are partially imprinted could reflect an adjustment of maternal and paternal allele expression to a new level of optimal total gene expression that relies on the mechanisms of gene expression regulation already in place from when the gene was expressed monoallelically. Interestingly, the kinship theory does predict that the expression of PEGs will be reduced as plants become self-fertilizing [15] and we find that partial imprinting appears to be more common for PEGs than MEGs (Table S6). The preponderance of MEGs over PEGs, regardless of partial vs. complete imprinting, also fits predictions of the maternal-offspring coadaptation theory of imprinting [16].

An alternative, non mutually exclusive, possibility is that the partially imprinted genes do not reflect a record of past conflict but are instead imprinted as a form of gene dosage regulation. Many of the imprinted genes encode transcriptional regulators and chromatin modifiers – proteins that function in macromolecular complexes that can be dosage sensitive. But why would dosage regulation be subject to parent-of-origin effects? It may be that these genes are taking advantage of existing molecular differences already tied to one parent – namely demethylation of the maternal genome before fertilization. Because the presence or absence of DNA methylation can influence gene expression levels, demethylation provides a built-in mechanism of dosage regulation that is specific to the parent-of-origin. We expect that the parent-of-origin specific effects on gene expression are due to some combination of parental conflict, maternal-offspring coadaptation, and dosage regulation, with different evolutionary pressures possibly acting at different loci. Genomic analysis of imprinting in outcrossing relatives of *A. thaliana* will help test these ideas.

## Supporting Information

Table S1 **Col/L** ***er*** **SNPs used in this study.**
(TXT)

Table S2 **Gene expression in the endosperm.**
(XLS)

Table S3 **Gene expression in the embryo.**
(XLS)

Table S4 **Expression of transposable elements in endosperm.**
(XLS)

Table S5 **Expression of transposable elements in embryo.**
(XLS)

Table S6 **Endosperm imprinted genes.**
(XLS)

Table S7 **Embryo imprinted genes.**
(XLS)

Table S8 **Validation of candidate imprinted genes from Gehring** ***et al.*** **2009.**
(XLS)

Table S9 **Subset of endosperm imprinted genes also identified as imprinted in Hsieh** ***et al.*** **2011.**
(XLS)

Table S10 **Analysis of Hsieh** ***et al.*** **read count data for LCMD pure endosperm using the statistical pipeline described in this study.**
(XLS)

Table S11 **Genes identified as imprinted when Hsieh** ***et al.*** **LCMD pure endosperm read count data is analyzed using the statistical criteria of this study.**
(XLS)

Table S12 **qPCR primers used in this study.**
(XLS)
